# Progress in sepsis prediction models: from traditional scoring systems to multimodal intelligence and clinical translation

**DOI:** 10.3389/fmed.2026.1732164

**Published:** 2026-02-11

**Authors:** Chenglin Mou, Jinbin Yang, Qingmei Wu, Lian Qin, Junyu Lu

**Affiliations:** 1 Intensive Care Unit, The Second Affiliated Hospital of Guangxi Medical University, Nanning, China; 2 Department of Emergency, Guigang City People's Hospital, Guigang, China; 3 Wuzhou Gongren Hospital, Wuzhou, China

**Keywords:** clinical decision support, deep learning, machine learning, multimodal data, prediction models, sepsis, translational medicine

## Abstract

Sepsis is a leading cause of mortality and healthcare expenditures among patients in the intensive care unit (ICU). Its pathophysiology is complex and its clinical manifestations are highly heterogeneous; early identification and timely, targeted interventions are essential to improving outcomes. With the widespread adoption of electronic health records (EHRs) and the rapid expansion of critical care data, developing sepsis prediction models using machine learning (ML) and deep learning (DL) has become an active area of research. This review provides a systematic overview of advances in sepsis prediction, from clinical problem framing and outcome definitions to data sources, feature engineering, and methodological evolution. We summarize the progression from traditional scoring systems (e.g., SOFA, qSOFA) to modern ML algorithms (e.g., gradient boosting trees, random forests) and time series DL models (e.g., LSTM, Transformer models). We also outline reporting and evaluation standards (e.g., TRIPOD AI), and synthesize evidence on representative models for early warning, prognostic risk stratification, and prediction of organ dysfunction. Key translational challenges are discussed, including generalization, fairness, model drift, workflow integration, alarm fatigue, and real world utility. Finally, we highlight opportunities in multimodal data fusion, causal inference, federated learning, and digital twins for building next generation, clinically actionable sepsis intelligence, and we offer practical recommendations to help move from algorithmic accuracy to demonstrable clinical value, emphasizing that only models that are externally validated, well calibrated, prospectively evaluated, and tightly aligned with clinical workflows are likely to improve patient outcomes.

## 1 Introduction

Sepsis is a life-threatening organ dysfunction caused by a dysregulated host response to infection. Recent global estimates indicate that sepsis remains a leading cause of mortality worldwide. Based on the latest Global Burden of Disease Study 2023, it is estimated that in 2021 there were approximately 166 million sepsis cases globally, resulting in about 21.4 million deaths, accounting for roughly one-third of all deaths worldwide. Previous estimates from 2017 suggested about 49 million incident sepsis cases with 11 million sepsis-related deaths, representing nearly 20% of all global deaths. These data underscore that sepsis continues to impose a substantial public health burden, particularly in low- and middle-income settings (1–3). The core pathophysiological feature is progressive multiple organ dysfunction syndrome (MODS), and once it progresses to septic shock, the in-hospital mortality rate sharply increases. Numerous pieces of evidence indicate that early identification and timely initiation of sepsis bundles centered on antimicrobial therapy and hemodynamic support are key to improving patient survival (4). However, the early symptoms of sepsis are often atypical and deceptive, especially in elderly, immunocompromised, or patients with multiple underlying diseases, which constitutes a key challenge in clinical practice: how to sensitively capture risk signals from massive and dynamic clinical information in the prodromal stage or when organ dysfunction is reversible.

The definition of sepsis has evolved from the Sepsis-1.0/2.0 standard based on systemic inflammatory response syndrome (SIRS) to the Sepsis-3 standard based on sequential organ failure assessment (SOFA) score (1). This shift emphasizes the central role of organ dysfunction in the diagnosis of sepsis, but also brings new challenges. The definition of “suspected infection” in Sepsis-3 lacks an objective and unified standard, and the calculation of SOFA score relies on multiple laboratory tests and clinical evaluations. Its operability varies in different studies and clinical scenarios. This has led to ambiguity and delay in the “gold standard” label for sepsis, directly affecting the development, validation, and comparison of predictive models.

In recent years, the widespread implementation of EHRs, critical care information systems (CIS), and bedside monitoring has transformed predictive research (5), enabling the discovery of complex nonlinear relationships from large, multicenter, high granularity real world data, including ICU databases (e.g., MIMIC, eICU) (6, 7), bedside high-frequency continuous monitoring streams (4), and unstructured clinical text and imaging. This has given rise to a large number of sepsis prediction models based on machine learning (ML) and deep learning (DL), which demonstrate distinct strengths in discrimination under appropriate data availability and validation settings, while also introducing higher requirements for data quality, implementation, and reproducibility compared with rule-based clinical scores.

However, despite significant technological advancements, the translation of these high-precision models into clinical practice has been difficult (8). Insufficient generalization, model fairness issues, lack of prospective impact studies, and difficulties in integrating with clinical workflows collectively constitute the “last mile” obstacles. This review aims to comprehensively review the current research status of sepsis prediction models, systematically elaborating on the entire process methodology from clinical problem definition, data preparation, model construction to evaluation and validation, with a focus on the paradigm shift from traditional statistical models to modern artificial intelligence methods. At the same time, we will delve into the key challenges faced by the clinical translation of the model and look forward to the future development direction of multimodal intelligence, causal inference, and integrated diagnosis and treatment, in order to provide reference for future research and practice in this field.

**Figure 1:**
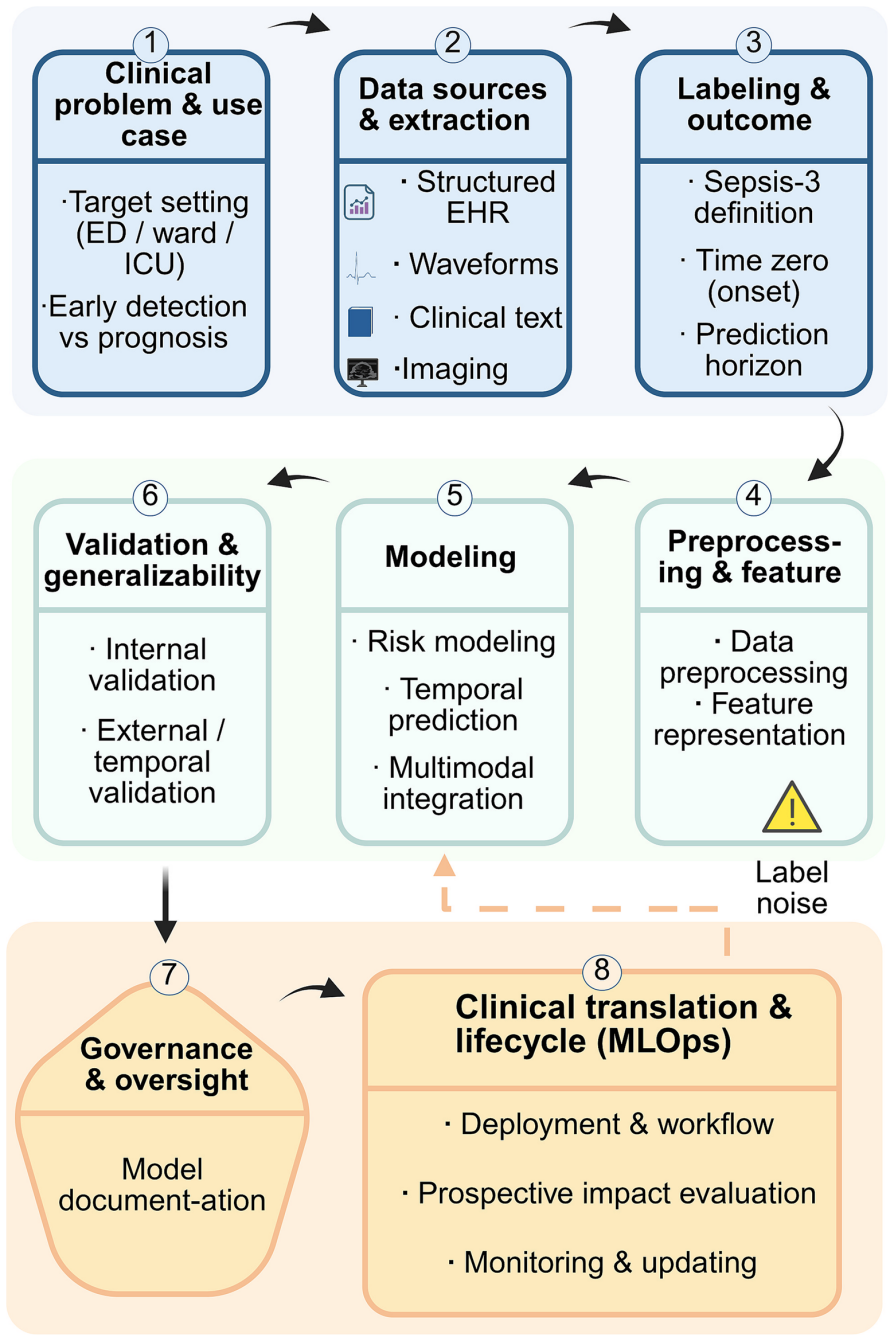
Methodological workflow and clinical translation framework for sepsis prediction models. This figure illustrates a comprehensive end-to-end framework for the development, validation, and real-world implementation of sepsis prediction models. The workflow begins with the definition of the clinical problem and use case, including care settings (emergency department, general ward, or ICU) and prediction objectives (early detection versus prognosis). Multiple data sources are subsequently integrated, encompassing structured electronic health records, physiological waveforms, clinical text, and imaging data. Outcome labeling is typically based on the Sepsis-3 definition, with explicit specification of time zero and prediction horizons; however, this step is inherently challenged by label noise and population heterogeneity. Data preprocessing and feature representation precede model development, which may range from traditional risk modeling approaches to temporal prediction and multimodal integration. Model performance and robustness are assessed through internal validation as well as external and temporal validation to evaluate generalizability. Beyond model development, the framework highlights governance and oversight, including model documentation, and emphasizes clinical translation through deployment, workflow integration, and prospective impact evaluation. Continuous monitoring, updating, and recalibration within an MLOps lifecycle are important considerations for maintaining model performance, safety, and clinical relevance after deployment.

Figure 1 provides a conceptual overview of the end-to-end paradigm underlying sepsis prediction research, highlighting the methodological continuum from problem formulation and model development to clinical translation and real-world implementation.

## 2 Clinical classification for prediction tasks and outcome setting

The clinical application scenarios of sepsis prediction models are diverse, and their value is reflected in decision support for different stages and problems. According to clinical needs, prediction tasks can be roughly divided into the following categories.

### 2.1 Early identification and onset prediction

This is the most active line of research, aiming to issue alerts hours before a definitive diagnosis of sepsis. Models are typically deployed in emergency departments (ED), general wards, or ICUs, with the goal of identifying infected or suspected infected patients at risk of progressing to sepsis (2). The setting of the lead-time prediction window is crucial, usually between 4 and 24 h (4, 9). An effective warning system must strike a balance between providing sufficient intervention time and avoiding excessively high false positive rates (9).

### 2.2 Prognostic prediction

Predicting short-term or long-term adverse outcomes for patients diagnosed with sepsis can aid in risk stratification, resource allocation, and treatment decision-making. Common outcomes include hospitalization/ICU mortality rate (5, 10), 28-day or 90-day mortality rate (11), as well as long-term functional outcomes such as post–intensive care syndrome (PICS).

### 2.3 Prediction related to complications and organ function

The harm of sepsis is mainly achieved by inducing organ dysfunction. Therefore, predicting the risk of specific organ complications, such as septic shock, has important clinical value (12), acute respiratory distress syndrome (ARDS), acute kidney injury (AKI) (13), disseminated intravascular coagulation (DIC) and sepsis associated encephalopathy (SAD) (14), etc.

### 2.4 Process based outcome prediction

This type of task focuses on the consumption of medical resources, such as ICU or length of hospital stay (LOS) (15), the risk of readmission and the need for mechanical ventilation or hemodynamic support.

### 2.5 Individualized treatment related predictions

This is a cutting-edge application of predictive models aimed at guiding treatment choices. For example, predicting patients’ responsiveness to fluid resuscitation and their demand for specific vasoactive drugs (16), or predict its response to immunomodulatory therapy based on immune phenotype.

When setting these outcomes, it is necessary to precisely define the time zero, observation window, and prediction window at which the event occurs (17). For binary classification tasks, determining the event time is crucial. In survival analysis, competitive risks (such as discharge) and censoring need to be considered (18). The setting of lead time for prediction needs to take into account clinical operability, that is, the reasonable time from the issuance of the alarm to the effective intervention of the clinical team. In addition, the definitions of all outcome measures should be standardized and operationalized as much as possible, for example, by uniformly adopting the Sepsis-3 standard, clarifying the hemodynamic definition of shock, and dynamically evaluating changes in organ function scores, which is crucial for ensuring the comparability and reproducibility of the model (19).

## 3 Data sources and feature space

The data sources for sepsis prediction models are becoming increasingly diverse, forming a multimodal feature space that provides a foundation for capturing complex disease signals.

### 3.1 Structured clinical data

This is the most commonly used type of data, sourced from EHR and CIS. It includes: Vital signs: such as heart rate, blood pressure, respiratory rate, body temperature, oxygen saturation, etc. (4). Laboratory examination: Complete blood count (CBC) (2, 20), blood gas analysis, liver and kidney function, coagulation indicators, etc. Medication records: antibiotics, vasoactive drugs, sedatives, etc. Fluid intake and output: Accurately recording the fluid balance is an important indicator for evaluating the circulation state. Clinical scoring: SOFA, APACHE II, SAPS II and their dynamic changes can serve as strong predictive factors (7).

### 3.2 Continuous signal and bedside waveform

The high-frequency data streams generated by bedside monitors, such as electrocardiogram (ECG) waveforms, invasive arterial pressure waveforms, ventilator parameter curves, etc., contain richer physiological information than sparsely recorded vital signs. Signal processing and feature extraction of these waveforms can capture heart rate variability (HRV) (21), the variability of blood pressure and subtle changes in cardiopulmonary interaction(s) may be early signals of organ dysfunction.

### 3.3 Unstructured text and images

*Clinical text*: Through natural language processing (NLP) technology, key information such as evidence of “suspected infection,” symptom descriptions, physical examination findings, etc. can be extracted from medical orders, course records, nursing records, and discharge summaries to compensate for the lack of structured data (22). *Medical imaging*: Deep learning, especially convolutional neural networks (CNN), can extract information from chest X-rays (CXR) (23), automatically extract imaging features from bedside ultrasound (POCUS) or CT scans for identifying the source of infection (such as pneumonia) (24) or evaluate organ damage (such as pulmonary edema, heart function) (25).

### 3.4 Multi omics and biomarkers

*Traditional biomarker*: procalcitonin (PCT) (20), C-reactive protein (CRP), interleukin-6 (IL-6), lactate, lactate and its clearance are important auxiliary tools for the diagnosis and prognosis of sepsis. Some studies have also explored novel biomarkers such as soluble urokinase type plasminogen activator receptor (suPAR) and lipids (such as ceramides) (26) predictive value. *Multi omics data*: High throughput techniques such as transcriptomics, proteomics, and metabolomics can reveal the complex host response network of sepsis. By identifying specific gene expression profiles or metabolite characteristics, patients can be stratified into different immune endotypes, providing the possibility for precise treatment and personalized prediction. *Microbiological data*: including pathogen identification, microbiome composition, and antibiotic resistance spectrum, are crucial for guiding anti infective treatment and predicting treatment response.

Data quality and governance are the foundation of successful modeling. When dealing with these multi-source heterogeneous data, many challenges must be addressed, including data loss (and its underlying mechanisms such as information loss), imprecise timestamps and alignment issues, delays in label acquisition, and multi center data heterogeneity caused by differences in practice among different medical institutions. Adopting standardized data models (such as OMOP-CDM) (27) and strict data governance processes are key to improving research quality and model portability.

### 3.5 Benchmark datasets and open challenges

To facilitate reproducibility and benchmarking, several large, publicly accessible datasets and benchmark challenges have shaped sepsis prediction research (Table 1).

**Table 1:** Key publicly available datasets and benchmark challenges for sepsis prediction.

Resource	Description	Typical use in sepsis research
MIMIC-III/MIMIC-IV (PhysioNet)	Single-center ICU electronic health record data with high temporal resolution	Model development, retrospective validation, time-series modeling
eICU Collaborative Research Database	Large multi-center ICU database across the United States	External validation, generalizability and transportability studies
PhysioNet/CinC Sepsis Challenge (2019)	Standardized benchmark dataset with shared sepsis labels and evaluation metrics	Comparative model evaluation under consistent definitions and metrics
HiRID, AmsterdamUMCdb	High-resolution European ICU datasets	Dynamic risk prediction, robustness analysis, and performance drift assessment

Location of revision: Section 3, Benchmark datasets and open challenges. Representative dataset overview publications are cited for commonly used ICU databases (54, 55).

## 4 Genealogy of model methodology

The technical roadmap of sepsis prediction models has evolved along a methodological continuum, encompassing clinical scoring systems, conventional machine learning, and increasingly complex deep learning–based approaches.

*Rule-based clinical scoring systems*: Rule-based clinical scoring systems include classic indices such as SIRS, qSOFA, SOFA, APACHE, and SAPS, as well as recently updated or revised tools such as NEWS2, the Helicopter Emergency Medical Service (HEMS) score, and laboratory-based indices like the LIP score. These scoring systems are constructed from a relatively limited set of clinical and/or laboratory variables and rely on predefined rules and simple calculations, enabling rapid bedside implementation. They have long been used for risk stratification and severity assessment in routine clinical practice. However, most rule-based scores are designed for population-level assessment and may have limited flexibility for early, dynamic, and individualized prediction across heterogeneous clinical settings, with performance that can vary depending on context and case mix (28). *Statistical regression–based models*: Logistic regression and Cox proportional hazards regression represent classic statistical approaches for predictive modeling. These methods offer strong interpretability and transparent parameter estimation but rely on assumptions such as linearity and proportional hazards, which may limit their ability to capture complex, nonlinear relationships in real-world clinical data (29). *Updated and revised clinical scoring systems*: In parallel, several rule-based clinical scores have been updated or newly proposed in recent years to address limitations of earlier tools. For example, NEWS2 and the HEMS score have been adopted in pre-hospital and emergency settings for early detection of clinical deterioration, while the LIP score, derived from routinely available laboratory parameters, demonstrated promising discriminatory performance for sepsis identification in a 2022 cohort (30). More recently, the SOFA-2 revision released in 2025 aimed to refine organ dysfunction assessment while preserving the interpretability and bedside applicability characteristic of clinical scoring systems. Despite these updates, such scores continue to balance simplicity and usability against limitations in sensitivity and individual-level precision.

### 4.1 Machine learning

*Common algorithms*: compared with traditional statistical models, machine learning algorithms can better capture nonlinear relationships and high-order interactions in data. Tree-based models, such as Random Forest (RF) (5), Extreme Gradient Boosting (XGBoost) (7, 31), and LightGBM (32), due to their high performance and robustness, are widely used in sepsis prediction. Other algorithms such as Support Vector Machine (SVM), K-Nearest Neighbor (kNN), and Naive Bayes are also commonly used as comparison baselines (10). *Feature engineering*: The success of machine learning largely relies on feature engineering, which involves extracting meaningful variables from raw data. This includes creating lag features, differential features, and statistical features that reflect physiological variability (such as standard deviation, coefficient of variation) to capture the dynamic evolution of diseases (33).

### 4.2 Deep learning and temporal modeling

*Temporal model*: For time series data collected intensively in ICU, deep learning models can automatically learn complex temporal dependencies (34). Recurrent Neural Networks (RNNs) and their variants—Long Short-Term Memory Networks (LSTM) (35) and Gated Recurrent Unit (GRU)—are commonly used architectures for processing such data. In recent years, Time Convolutional Networks (TCNs) and Transformer models based on self attention mechanisms have also received attention due to their advantages in processing long sequence data. *Representation learning*: Another major advantage of deep learning is the ability to perform self supervised learning from unlabeled data, resulting in rich data representations. For example, models can be trained from massive ECGs (36) or learn universal physiological state representations from arterial pressure waveforms, and then use these representations for downstream sepsis prediction tasks (37). *Multimodal Fusion*: Deep learning frameworks provide powerful capabilities for fusing heterogeneous data such as structured data, text, images, and waveforms. By designing specific network structures (such as attention mechanisms) to integrate information from different modalities, it is expected to achieve more accurate predictions than a single data source (38).

### 4.3 Cause and effect and individualization

*Causal inference*: Traditional prediction models mainly focus on correlation, while clinical decision-making requires more causal insights. The causal inference method aims to estimate the individualized treatment effects (ITE) of treatment interventions from observational data to avoid confounding bias caused by treatment choices themselves (39). *Reinforcement Learning (RL)*: RL provides a theoretical framework for sequential decision problems. By modeling patient status, clinical interventions, and long-term outcomes as a Markov decision process, RL can learn optimal dynamic treatment strategies, such as fluid management or titration of vasoactive drugs, achieving the integration of prediction and decision-making (40).

### 4.4 Federated learning and privacy protection

In multi center collaboration, data privacy and security are the main obstacles. Federated Learning allows centers to collaboratively train a global model by exchanging model parameters without sharing raw patient data, providing a feasible solution for building large-scale and diverse prediction models (41).

To facilitate comparison across paradigms, Table 2 summarizes key characteristics, typical use cases, and advantages and limitations of traditional scoring systems, conventional machine learning models, and deep learning approaches in sepsis prediction.

**Table 2:** Summary of methodological paradigms for sepsis prediction.

Paradigm	Typical examples	Data requirements	Strengths	Limitations / Challenges	Typical use cases
Traditional clinical scores	SIRS, qSOFA, SOFA, APACHE II, SAPS II, NEWS/NEWS2, HEMS, LIP score	Small set of bedside and lab variables	Simple, transparent, widely understood; minimal infrastructure	Limited personalization; often static; may be poorly calibrated for early prediction	Triage and initial risk stratification in ED/wards (1, 56)
Conventional ML models	Logistic regression with feature engineering, RF, XGBoost, LightGBM, SVM	Structured EHR/CIS data; dozens–hundreds of features	Captures nonlinearity; flexible; can be well calibrated with careful training	Feature engineering burden; domain shift; deployment and monitoring required	Early warning, mortality risk, organ dysfunction prediction (4, 52)
Deep learning / temporal models	RNN/LSTM/GRU, TCN, Transformers	High-frequency time series; multimodal (signals/text/images)	Representation learning; strong temporal modeling; multimodal fusion	Data/compute intensive; interpretability and deployment complexity; overfitting risk	Real-time ICU monitoring; multimodal decision support (16)

Location of revision: After Section 4 (“Federated Learning and Privacy Protection”).

**Figure 2:**
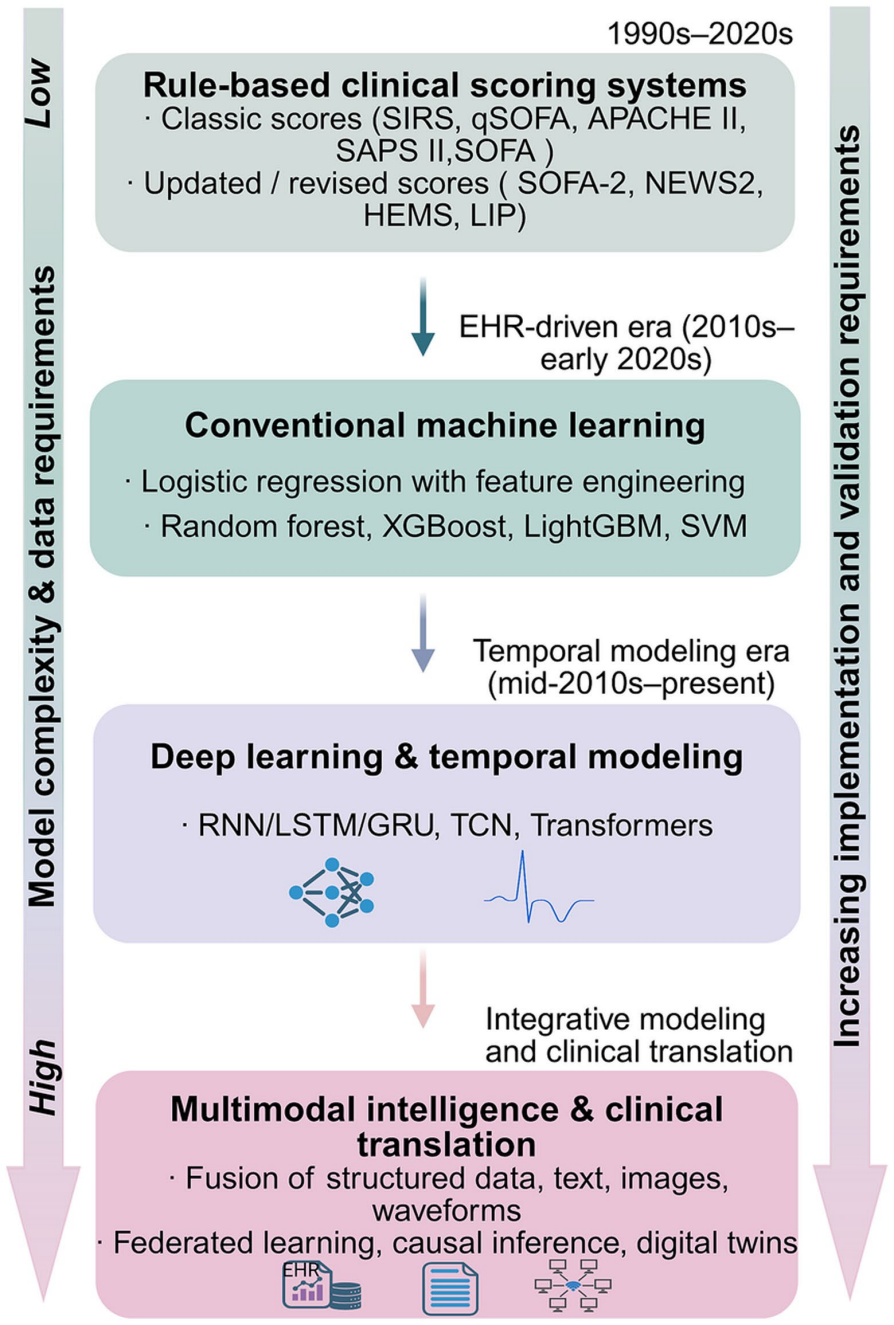
Methodological spectrum of sepsis prediction approaches across clinical scoring, machine learning, and multimodal models. The figure is organized into four modules and summarizes major methodological paradigms that have been applied to sepsis prediction, ranging from rule-based clinical scoring systems to multimodal approaches. Rule-based clinical scoring systems include classic scores (e.g., SIRS, qSOFA, APACHE II, SAPS II, SOFA) as well as updated or revised indices (e.g., SOFA-2, NEWS2, the Helicopter Emergency Medical Service [HEMS] score, and the LIP score), which emphasize bedside interpretability using relatively limited sets of clinical and/or laboratory variables. Subsequent paradigms incorporate structured electronic health record data and conventional machine learning methods, such as logistic regression with feature engineering and tree-based or kernel-based algorithms, enabling more flexible risk modeling. More recent developments apply deep learning and temporal modeling techniques to high-resolution ICU time-series data, supporting dynamic and continuous risk estimation. Emerging multimodal approaches further integrate heterogeneous data sources, including structured variables, clinical text, medical imaging, and physiological waveforms, while introducing increased requirements for validation, implementation, and clinical translation. The vertical arrangement reflects increasing data heterogeneity and model complexity, as well as rising demands for validation and deployment, and does not imply hierarchical clinical superiority among paradigms.

Figure 2 provides an overview of major methodological paradigms in sepsis prediction, spanning rule-based clinical scores, conventional machine learning, deep learning with temporal modeling, and emerging multimodal approaches.

## 5 Model development process and evaluation standards

It is crucial to follow strict development, evaluation, and reporting standards to ensure the scientific and reliable nature of sepsis prediction models.

### 5.1 Research design and cohort construction

*Clear definition*: It is necessary to clarify the inclusion and exclusion criteria of the study, the precise operational definition of events (such as sepsis onset), and the time zero (such as ICU admission time or the time when the suspected infection criteria are first met). *Sample size*: Sufficient sample size and number of events should be ensured to avoid overfitting of the model. *Data partitioning*: The dataset should be strictly divided into training set, validation set, and testing set. The most ideal validation method is to conduct external validation in time and space, that is, to test the performance of the model on data from different time periods (temporal external validation) or different medical centers (external validation across centers), in order to evaluate its generalization ability (7, 20).

### 5.2 Handling of deficiencies and imbalances

*Missing data*: The proportion and pattern of missing data should be reported and processed using appropriate methods such as multiple imputation (42). Some models, such as gradient boosting trees, can inherently handle missing values. Sometimes, the absence itself is a form of information (informative missingness) that can be used as a feature input model. *Category imbalance*: Adverse events such as sepsis are usually rare events in the population, which can lead to imbalanced data categories. The processing methods include resampling (oversampling or undersampling) or introducing category weights in the loss function. When evaluating model performance on imbalanced datasets, the area under the precision recall curve (AUPRC) is typically more informative than the area under the receiver operating characteristic (ROC) curve (43).

### 5.3 Performance and calibration evaluation

*Discrimination*: measures the ability of a model to distinguish between patients with and without events, commonly measured by AUROC and AUPRC (44). *Calibration*: Evaluating the consistency between the predicted probability of the model and the actual observed frequency. A well calibrated model that predicts a 20% risk should correspond to approximately 20% of patients in that risk group actually experiencing events (45). The evaluation tools include calibration curve, Brier score, and calibration slope. *Clinical utility*: Decision curve analysis (DCA) is an effective tool for evaluating the clinical net benefits of a model. It links the predictive performance of the model to specific decision thresholds, helping clinicians determine at which risk threshold to use the model more beneficial than the default strategy of “treat all” or “not treat all” (46).

### 5.4 Preventing information leakage and bias

*Data leakage*: in the process of model development, it is necessary to be vigilant about the possibility of future information leakage to past predictions (47). For example, using data after an event to train a model that predicts events before they occur. When processing time series data, the division of cross validation should maintain temporal continuity. *Common bias*: it is necessary to pay attention to and try to control the backdoor bias and immortal time bias caused by intervention measures.

### 5.5 Transparency and reporting

*Reporting guidelines*: Research reports should follow internationally recognized guidelines, such as TRIPOD (Transparent Reporting of Multivariate Prediction Models) (48)And its extension TRIPOD-AI for AI models, as well as PROBAST (a bias risk assessment tool for predictive model research). *Reproducibility*: In order to promote scientific validation and collaboration, researchers should make their code, model weights, and provide detailed data dictionaries and model cards as much as possible to enhance the reproducibility of their research.

## 6 Representative models and evidence summary

In recent years, a large number of sepsis prediction models based on machine learning and deep learning have been developed and shown potential in different tasks.

### 6.1 Early warning/outbreak prediction model

The “Artificial Intelligence Sepsis Expert” (AISE) algorithm developed by Nemati et al. (4) utilizes 65 clinical variables to predict sepsis attacks 4 to 12 h in advance in the ICU, demonstrating good performance on an external validation set. SepsisAI developed by Gupta et al. (35), a deep learning model based on LSTM, aims to predict in-hospital sepsis in real-time and has specially designed alarm suppression logic to reduce alarm fatigue. Some studies focus on general ward or emergency department scenarios, using more limited datasets for early warning, which is crucial for preventing patients from deteriorating and being transferred to the ICU. These models typically require a trade-off between early warning and positive predictive value (PPV) to adapt to the resources and workflows of different clinical environments.

### 6.2 Prediction of death and severe outcomes

Many studies have shown that machine learning models outperform traditional scoring in predicting mortality rates in sepsis patients. For example, Taylor et al. (5) used a random forest model to predict in-hospital mortality in emergency sepsis patients, which performed better than traditional methods. The XGBoost based model developed by Wang et al. (7) showed excellent mortality prediction ability in both MIMIC-IV database and external validation of Chinese teaching hospitals (AUROC were 0.873 and 0.844, respectively), and provided interpretability analysis based on SHAP. Li et al.’s (10) study also confirmed that gradient boosting decision trees (GBDT) perform well in predicting mortality rates in ICU sepsis patients.

### 6.3 Prediction of organ function and complications

Progress has also been made in predictive models for specific organ dysfunction. For example, Zhang et al. developed an integrated machine learning model for early prediction of sepsis related AKI (13), and another study focused on predicting sepsis associated delirium (14). These models help clinical doctors take preventive measures in advance.

### 6.4 Biomarkers and integrated multi omics models

Research has shown that combining routine laboratory tests such as CBC with machine learning can effectively predict sepsis without relying on expensive or delayed biomarkers (2, 20). The model that integrates multiple omics data is still in the exploratory stage, but has shown great potential. By identifying transcriptome features associated with specific immune response patterns, patients can be stratified for risk and may guide future immunomodulatory therapies.

### 6.5 Summary

Existing research has shown that machine learning and deep learning models typically achieve high levels of discrimination in sepsis prediction tasks (with AUROC mostly between 0.80–0.95). Compared to traditional scoring, these models can better utilize massive and dynamic data. However, many models still lack rigorous external validation, and their transferability in different populations and medical environments remains a key issue. In addition, the complexity of the model also requires interpretability so that clinical doctors can understand and trust its predictive results.

## 7 Generalization, fairness, and model updating

A model that performs well on single center data may experience significant performance degradation in new clinical environments. This is an obstacle that the model must overcome in its clinical application, involving multiple dimensions such as generalization, fairness, and continuous maintenance.

### 7.1 Domain shift and time drift

The main reason for the decline in model performance is the change in data distribution, known as “domain shift.” This can be a spatial shift between different hospitals or regions, or a temporal drift caused by changes in clinical practice, testing equipment, patient populations, or disease epidemiology (such as the COVID-19 pandemic) over time (6, 49). Therefore, strict external validation and temporal external validation are the gold standards for evaluating model generalization.

### 7.2 Re calibration and adaptation strategies

When the model is deployed to a new environment, even if its discrimination remains good, its calibration may deteriorate. Therefore, it is necessary to recalibrate the model, such as adjusting its prediction probability through methods such as Platt scaling or isotonic regression. More advanced strategies include transfer learning and domain adaptation, which can utilize a small amount of labeled or unlabeled data from new environments to fine tune the model to adapt to new data distributions.

### 7.3 Fairness and subgroup performance

A model that performs well in the overall population may perform poorly in specific subgroups (such as patients of different ages, genders, races, or with specific underlying diseases). This may be due to systematic bias present in the data. Therefore, it is necessary to evaluate the fairness of the model’s performance in key subgroups to ensure that it does not exacerbate healthcare inequality. For example, in scenarios of immune suppression, elderly vulnerability, or resource constraints, the performance of the model may require special attention.

### 7.4 Monitoring and MLOps

Model deployment is not a one-time solution. A machine learning operations (MLOps) system needs to be established to continuously monitor the online performance of the model and detect drift in its discrimination and calibration. Once performance degradation is detected beyond an acceptable range, an alert needs to be triggered and the model’s rollback, retraining, or update process needs to be initiated to form a closed-loop model governance and lifecycle management.

## 8 Clinical translation and real world utility evaluation

Translating a technically validated predictive model into a clinical tool that can improve patient outcomes is a complex multi-step process. In practice, the clinical translation of sepsis prediction models is constrained by multiple, interrelated barriers that extend beyond model discrimination performance alone. These challenges span data and label quality, model generalizability and temporal drift, workflow integration and alarm fatigue, interpretability and clinician trust, regulatory and governance considerations, as well as the scarcity of prospective impact evidence. To provide a structured and implementation-oriented overview, the key barriers to clinical translation and their corresponding mitigation strategies are summarized in Table 3.

**Table 3:** Key barriers to clinical translation of sepsis prediction models and proposed mitigation strategies.

Key barrier	Core issue	Mitigation strategies	Representative evidence
Data quality and label heterogeneity	Inconsistent sepsis definitions and noisy or delayed labels across EHR and administrative data.	Standardized operational definitions (e.g., Sepsis-3); transparent labeling pipelines; data quality audits.	Singer et al., JAMA, 2016 (56); Karlic et al., Ann Am Thorac Soc 2023 (57)^.^
Generalizability and performance drift	Performance degradation across sites or over time due to distributional shift.	Prospective external and temporal validation; drift monitoring; scheduled recalibration.	Parikh et al., JAMIA, 2023 (58).
Workflow integration and alarm fatigue	Poor alert integration and high false-positive rates reduce clinician trust and adoption.	Clinician co-design; tiered and context-aware alerts; silent-mode or pilot deployment.	Zhang et al., NPJ Digital Medicine, 2022 (59);
Interpretability and algorithmic bias	Black-box perception, automation bias, and unequal subgroup performance.	Patient-level explanations (e.g., SHAP); subgroup audits; bias-aware evaluation.	Chen et al., Nat Biomed Eng 2023 (60); Weng et al., Lancet Digital Health, 2024 (61).
Regulatory and governance challenges	Unclear oversight pathways for adaptive AI/ML medical devices.	Lifecycle governance frameworks; explicit documentation; postmarket surveillance.	Muehlematter et al., Lancet Digital Health, 2023 (62); Babic et al., NPJ Digital Medicine, 2025 (63).
Evidence gap in real-world impact	Limited prospective or randomized evidence of clinical benefit.	Pragmatic trials; cluster or stepped-wedge RCTs; standardized impact reporting.	Adams et al., Nature Medicine, 2022 (64); Arabi et al., JAMA, 2024 (65).

Location of insertion: Section 8 “Clinical Translation and Real World Utility Evaluation”, immediately after the introductory paragraph that outlines the multiple interrelated barriers to real-world deployment.

### 8.1 Workflow integration

The model must be seamlessly integrated into existing clinical information systems (such as EHR) and workflows. This involves how to present predictive results (such as risk scores, warning information), triggering logic, frequency, and suppression strategies of alerts to clinical doctors. A core challenge is to avoid ‘alarm fatigue’, where too many false positive alarms lead to clinical doctors losing trust and response to them (35).

### 8.2 Threshold and resource constraints

Any warning system needs to set a risk threshold for triggering actions. The selection of this threshold not only depends on the performance of the model (such as sensitivity and specificity), but should also be coupled with clinical resources (such as ICU beds, manpower). Through sensitivity analysis, it is possible to evaluate how the optimal threshold changes under different resource constraints to achieve the maximization of clinical net benefits.

**Figure 3:**
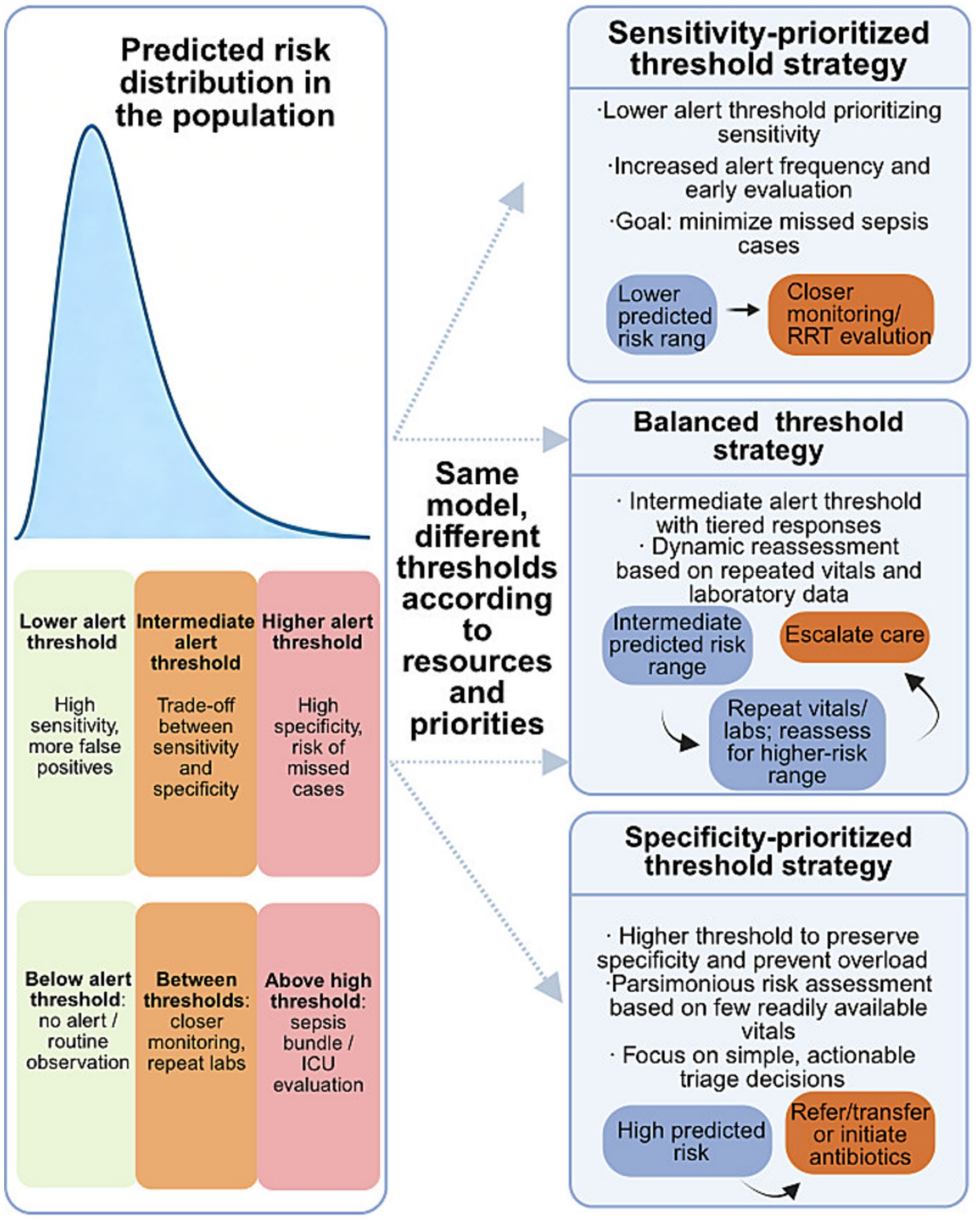
Relationship between predicted sepsis risk, alert thresholds, and clinical actions. This schematic illustrates how the same sepsis prediction model can support different clinical decision strategies through adaptive threshold selection. Predicted risk scores are distributed across the patient population, and different alert thresholds correspond to distinct trade-offs between sensitivity and specificity. Under sensitivity-prioritized strategies, lower thresholds favor early detection at the cost of increased false-positive alerts, enabling closer monitoring or early evaluation. Balanced strategies adopt intermediate thresholds with dynamic reassessment based on repeated vital signs and laboratory data. In contrast, specificity-prioritized strategies apply higher thresholds to preserve clinical resources and reduce alert burden, focusing on high-risk patients who warrant immediate escalation of care. This schematic illustrates how threshold selection may vary according to model performance, clinical resource availability, workflow capacity, and local clinical priorities, highlighting a decision-analytic perspective for translating prediction outputs into actionable bedside decisions.

Figure 3 schematically links model-predicted risk thresholds with resource constraints and potential clinical actions, highlighting how decision-analytic thinking can guide threshold selection.

Although workflow integration and threshold selection are essential, the real-world safety of sepsis prediction models ultimately relies on how clinicians interpret and act on model outputs. The following section summarizes clinician-oriented interpretive prerequisites for safe deployment.

#### 8.2.1 What clinicians need to know to safely use sepsis prediction models

The safe clinical use of sepsis prediction models requires not detailed knowledge of model construction, but a minimum set of clinician-oriented interpretive competencies.

First, clinicians should recognize the distinction between discrimination (e.g., AUROC) and calibration, as well-calibrated risk estimates are essential for meaningful bedside decisions that depend on absolute risk rather than ranking alone.

Second, the clinical meaning of risk thresholds should be understood as context-dependent trade-offs between sensitivity and specificity, shaped by care setting, resource availability, and clinical priorities, rather than fixed properties of a given model.

Third, clinicians should anticipate the presence of false-positive alerts as an inherent feature of early warning systems, with direct implications for alert fatigue, trust, and workflow burden.

Together, these elements define a pragmatic, minimum AI literacy framework for clinicians, supporting the responsible integration of sepsis prediction models as decision-support tools rather than autonomous decision-makers.

### 8.3 Impact research design

The ultimate evidence to prove the clinical utility of the model comes from prospective impact studies. *The research design can include*: pre - and post control study: comparing the changes in clinical process indicators (such as antibiotic initiation time, fluid resuscitation volume) and clinical outcomes (such as mortality rate, ICU length of stay) before and after model deployment. *Cluster randomized controlled trials (cRCTs)*: Randomly allocate medical units (such as wards or hospitals) to intervention groups (using models) and control groups (conventional treatments), which is the gold standard design for evaluating the effectiveness of clinical decision support systems (CDSS). *A/B testing or silent mode deployment*: Running the model in the real world without displaying the results to clinical doctors (silent mode) as a control to evaluate its potential impact. Although such research evidence is still scarce, some studies have begun prospective validation (50).

### 8.4 Experience and lessons learned from deployed systems

The experience of a few sepsis warning systems already deployed in clinical settings, such as Epic’s Sepsis Prediction Model, suggests that their real-world performance may be lower than reported during the development phase (28), this highlights the importance of continuous performance monitoring and external recalibration. In addition, the design of human-computer interaction, interpretability of models, and how to avoid automation bias (i.e., excessive reliance on models while ignoring clinical judgments) are key factors determining the success or failure of the system.

### 8.5 Economic evaluation

In addition to clinical outcomes, the economic impact of model deployment should also be evaluated, including changes in healthcare costs, resource utilization efficiency, and bed turnover, and a cost–benefit analysis should be conducted.

## 9 Special populations and specific situations

The pathophysiology and clinical manifestations of sepsis vary depending on the population and context, requiring specific predictive models.

### 9.1 Pediatrics and newborns

The physiological parameters and response patterns to infection in children and newborns are significantly different from those in adults. Data sparsity is the main challenge in modeling in this field. Meeus et al. developed a machine learning model for premature infants to predict late onset sepsis (LOS) and necrotizing enterocolitis (NEC), demonstrating promising potential (31). In pediatrics, age-dependent vital sign norms and different immune-response trajectories often require age-stratified modeling, pediatric-specific feature normalization, or explicit incorporation of age as a non-linear effect modifier. In neonates, models commonly integrate gestational age, birth weight, and ventilator parameters and are designed to function under sparse sampling and frequent missingness typical of NICU workflows.

### 9.2 Special adult subgroups

The risk and manifestations of sepsis vary among elderly and vulnerable populations, pregnant and postpartum women, as well as immunocompromised patients (such as tumors and organ transplants). For example, Ke et al. (51) developed an in-hospital mortality prediction model specifically for elderly sepsis patients. For immunocompromised patients, baseline inflammatory markers and vital signs may be blunted, making fixed thresholds less informative. Practical adaptations include emphasizing trajectory features (e.g., within-patient change), incorporating immunosuppression-related variables (e.g., neutropenia status, recent chemotherapy/transplant), and using subgroup-specific recalibration to reduce systematic underestimation or overestimation of risk.

### 9.3 Specific clinical contexts

Postoperative, trauma (9), and burn patients often have strong non infectious inflammatory reactions, which makes the diagnosis and prediction of sepsis more challenging, requiring models that can distinguish between infectious and non infectious inflammation.

### 9.4 Resource constrained areas and pre hospital scenes

In these environments, the available data (such as laboratory tests) is very limited. Therefore, it is necessary to develop simplified models based on a small number of easily accessible variables, such as vital signs and physical examinations, which can be implemented on low-cost hardware or even paper-based tools. Parsimonious models and early warning scores can be paired with structured triage protocols to support escalation decisions, while the development of wearable devices and remote monitoring technology provides new possibilities for scalable early warning in these scenarios.

Across these special populations, effective adaptation strategies consistently include subgroup-specific recalibration, greater reliance on within-patient trajectories rather than absolute thresholds, and explicit incorporation of context-specific constraints (e.g., data sparsity or resource limitation) into model design and evaluation.

## 10 Frontier directions and future trends

The research on sepsis prediction models is developing towards a more intelligent, accurate, and integrated direction.

### 10.1 Multimodal basic models and self supervised learning

Drawing on the success of NLP and computer vision, developing multimodal foundational models capable of processing and integrating clinical text, images, waveforms, and structured data will be an important direction for the future. By performing self supervised learning on massive unlabeled clinical data, these models can learn universal and rich physiological and pathological representations, providing a powerful foundation for various downstream prediction tasks.

### 10.2 Causal mechanism fusion and digital twin

Future models will not only predict ‘what will happen’, but also answer ‘why it will happen’ and ‘what if…’. By combining machine learning with causal inference and systems biology models, a “digital twin” that simulates the physiological state of patients can be constructed to conduct counterfactual inference, evaluate the potential impact of different intervention measures on individual patients, and achieve a leap from correlation prediction to actionable individualized decision support.

### 10.3 Immune phenotype and phenotype driven treatment decisions

Integrating multiple omics data into a predictive model aimed at identifying different immune subtypes of sepsis. This is expected to achieve “diagnosis and treatment integration”, where the model can predict high risks while also recommending treatment strategies targeting specific immune phenotypes (such as immune enhancement or immune suppression), and serve as a companion diagnostic tool to guide clinical trials and personalized treatments.

### 10.4 Continuous learning and compliance updates

In order to cope with model drift, future clinical AI systems need to have the ability of continuous learning, which means continuously learning and adapting from new data streams without completely forgetting old knowledge. This requires finding a balance between the automation of model updates and clinical validation, regulatory compliance, and may require exploring innovative models such as “regulatory sandboxes.”

### 10.5 Privacy and compliance

With the increasing demand for data sharing and model collaboration, technologies and regulations that protect patient privacy will become increasingly important. Privacy computing technologies such as federated learning and differential privacy, as well as compliance with regulations such as the Medical Device Software (SaMD) and the EU Artificial Intelligence Act, will be necessary conditions for model development and deployment.

### 10.6 Open collaboration and benchmarking

By sharing datasets (such as PhysioNet Challenge) (52), establishing reproducible baseline models and evaluation criteria can promote the healthy development of the field. Building an interdisciplinary team consisting of clinical experts, data scientists, and engineers is key to ensuring the clinical relevance and usability of the model.

## 11 Discussion

Despite significant progress in the research of sepsis prediction models, the path from high AUROC to clinical benefits remains challenging.

### 11.1 The conversion dilemma of high AUROC models

Many models that perform well on retrospective data are difficult to generate practical value in clinical practice due to complex and diverse reasons. Firstly, the evaluation criteria of the model may be too low (such as compared to simple clinical scoring), or there may be bias in label definition (such as based on coding for sepsis diagnosis). Secondly, high AUROC does not directly translate into clinical usability; the calibration degree of the model, performance at specific decision thresholds (PPV/NPV), and compatibility with clinical workflows are more important. Finally, human factors such as the acceptance and trust of clinical doctors, as well as alarm fatigue, are the ultimate checkpoints that determine whether technology can be implemented (8).

### 11.2 Rethinking interpretability in critical care models

In critical care medicine, the “black box” model often causes concerns among clinical doctors. Interpretability is not just about providing feature importance ranking. A more valuable explanation would be individualized and dynamic, able to tell clinical doctors why the model is sounding an alarm for this specific patient at the current time point. Techniques such as SHAP (7) or LIME can provide local explanations. Furthermore, providing counterfactual explanations (such as’ if a patient’s certain indicator changes, the predicted results will change ‘) may have more clinical guidance significance (53).

### 11.3 The value and position of traditional scoring

Although machine learning models usually outperform in performance, traditional scoring systems still have irreplaceable value in resource limited or fast evaluation scenarios due to their simplicity, transparency, and lack of complex computing environments. The future trend may be to adopt a hybrid strategy, such as using traditional scoring for initial screening, and then using computationally intensive machine learning models for fine evaluation of high-risk populations, or integrating traditional scoring as one of the features into machine learning models.

### 11.4 The minimum feasible roadmap for research and practice

For research teams or medical institutions wishing to develop and apply sepsis prediction models, a pragmatic roadmap should start with rigorous single center retrospective model development, followed by rigorous multi center, out of time external validation to confirm its robustness. On this basis, small-scale prospective silent deployment pilots can be conducted to evaluate their performance in the real world and potential impact on clinical workflows, and ultimately consider conducting larger scale impact studies.

#### 11.4.1 Limitations of current evidence

Most sepsis prediction studies to date are retrospective analyses of single-center databases, with models developed and evaluated on historical EHR data. This raises several concerns. First, sepsis labels are often generated using proxy operational criteria (e.g., combinations of cultures, antibiotics, and SOFA changes), introducing labeling noise and potential misalignment with bedside diagnoses. Second, optimism bias can occur when models are evaluated on datasets closely related to development data, even with cross-validation. Third, prospective impact studies and randomized trials demonstrating improvements in patient-centered outcomes remain scarce. Therefore, the current evidence base is stronger for technical performance than for causal impact on mortality, morbidity, or resource utilization, underscoring the need for prospective, methodologically rigorous evaluations.

## 12 Conclusion

Research on sepsis prediction models has evolved from traditional scoring systems to machine learning and deep learning approaches capable of integrating large-scale, multimodal clinical data. These models have demonstrated superior performance in retrospective studies for early warning and outcome prediction, yet their translation into tangible clinical benefit remains limited. The major challenge has shifted from building high-AUROC models to developing clinically usable, interpretable, and equitable systems that can be seamlessly integrated into healthcare workflows.

From a practical standpoint, a concrete roadmap for the next phase of work should include: (i) data standardization and governance, including interoperable data models and reproducible, operationalized sepsis labels; (ii) interdisciplinary collaboration between clinicians, data scientists, engineers, and regulators throughout the model lifecycle; and (iii) explicit consideration of regulatory and medico-legal requirements for deployment and for any continuously learning clinical AI system. Future studies should emphasize rigorous external and temporal validation, transparent reporting following TRIPOD and TRIPOD-AI guidelines, and prospective impact evaluations in real-world settings, ideally using pragmatic and randomized designs where feasible.

Ultimately, transforming predictive accuracy into improved patient outcomes requires not only technical innovation but also clinical relevance, ethical and fair model behavior across subgroups, robust governance of model updates and drift, and continuous evaluation of utility across different healthcare systems and resource settings.
